# Connexin 30 deficiency attenuates A2 astrocyte responses and induces severe neurodegeneration in a 1-methyl-4-phenyl-1,2,3,6-tetrahydropyridine hydrochloride Parkinson’s disease animal model

**DOI:** 10.1186/s12974-018-1251-0

**Published:** 2018-08-13

**Authors:** Atsushi Fujita, Hiroo Yamaguchi, Ryo Yamasaki, Yiwen Cui, Yuta Matsuoka, Ken-ichi Yamada, Jun-ichi Kira

**Affiliations:** 1 Department of Neurology, Neurological Institute, Graduate School of Medical Sciences, Kyushu University, 3-1-1 Maidashi, Higashi-ku, Fukuoka, 812-8582 Japan; 2 Physical Chemistry for Life Science Laboratory, Faculty of Pharmaceutical Sciences, Kyushu University, Fukuoka, 812-8582 Japan

**Keywords:** Parkinson’s disease, Dopaminergic neuron, Astrocyte, Connexin 30, MPTP

## Abstract

**Background:**

The first pathology observed in Parkinson’s disease (PD) is ‘dying back’ of striatal dopaminergic (DA) terminals. Connexin (Cx)30, an astrocytic gap junction protein, is upregulated in the striatum in PD, but its roles in neurodegeneration remain elusive. We investigated Cx30 function in an acute PD model by administering 1-methyl-4-phenyl-1,2,3,6-tetrahydropyridine (MPTP) to wild-type (WT) and Cx30 knockout (KO) mice.

**Methods:**

On days 1 and 7 after MPTP administration, we evaluated changes in astrocytic Cx30, Cx43, glial fibrillary acidic protein, and ionised calcium-binding adapter molecule 1 expression by immunostaining and biochemical analysis. Loss of DA neurons was evaluated by tyrosine hydroxylase immunostaining. Gene expression was analysed using A1, A2, pan-reactive astrocyte microarray gene sets, and M1, M2, and M1/M2 mixed microglial microarray gene sets. Real-time PCR and in situ hybridisation were performed to evaluate glial cell-derived neurotrophic factor (*Gdnf*) and *S100a10* expression. Striatal GDNF protein levels were determined by enzyme-linked immunosorbent assay.

**Results:**

MPTP treatment induced upregulation of Cx30 and Cx43 levels in the striatum of WT and KO mice. DA neuron loss was accelerated in Cx30 KO compared with WT mice after MPTP administration, despite no change in the striatal concentration of methyl-4-phenylpyridinium^+^. Astrogliosis in the striatum of Cx30 KO mice was attenuated by MPTP, whereas microglial activation was unaffected. Microarrays of the striatum showed reduced expression of pan-reactive and A2 astrocyte genes after MPTP treatment in Cx30 KO compared with WT mice, while M1, M2, and M1/M2 mixed microglial gene expression did not change. MPTP reduced the number of striatal astrocytes co-expressing *Gdnf* mRNA and S100β protein or *S100a10* mRNA and S100β protein and also reduced the level of GDNF in the striatum of Cx30 KO compared with WT mice.

**Conclusions:**

These findings indicate that Cx30 plays critical roles in astrocyte neuroprotection in an MPTP PD model.

**Electronic supplementary material:**

The online version of this article (10.1186/s12974-018-1251-0) contains supplementary material, which is available to authorized users.

## Background

Parkinson’s disease (PD) is a common neurological disorder characterised by progressive degeneration of dopaminergic (DA) neurons and the formation of cytoplasmic inclusions called Lewy bodies in the substantia nigra pars compacta (SNc). The resulting disruption of DA neurotransmission in the basal ganglia produces progressive extrapyramidal motor symptoms. A range of pathogenic mechanisms causing DA neuronal death has been proposed [1]. Recently, accumulating evidence suggests important roles for non-neuronal cells, especially astrocytes, in DA neuron degeneration [2].

Astrocytes are the most abundant glial cell type in the central nervous system and play crucial roles in brain homeostasis, providing metabolic, electrical, and structural support for surrounding neurons in both normal and pathological conditions [3]. Astrocytes are heterogeneous in their functions and morphologies, depending on their location, subtype, and developmental stage [4]. Following pathological brain insult, astrocytes undergo a dynamic transformation called reactive astrogliosis. The functions of reactive astrocytes are controversial, and they have been reported to play both neuroprotective and neurodegenerative roles, providing another example of their heterogeneity [5]. The roles of astrocytes largely depend on the molecules they release into and take up from the extracellular space. Recently, it has been proposed that two types of reactive astrocytes, harmful A1 and protective A2 types, should be recognised based on genetic classification [6, 7].

In PD, astrocytes accumulate α-synuclein in their cytoplasm, and the distribution of such cells parallels that of Lewy bodies [8]. Animal PD models have shown that the accumulation of α-synuclein aggregates in astrocytes promotes their secretion of proinflammatory cytokines and chemokines, resulting in microglial activation [9, 10]. In contrast, astrocytes exert neuroprotective functions by releasing a variety of trophic factors, such as glial cell-derived neurotrophic factor (GDNF) [11] and brain-derived neurotrophic factor (BDNF) [12]. Thus, because astrocytes can both facilitate and prevent neuronal damage, their precise roles in PD remain uncertain.

Striatal DA terminal loss is an early and dominant feature of PD, suggesting that PD pathology may begin in the terminals and progress retrogradely to neuron bodies in the SNc [13]. However, mechanisms for this retrograde degeneration (‘dying back’) are not well characterised. Studies in a 6-hydroxydopamine (6-OHDA)-induced rat model of PD showed that levels of Cx30, but not of Cx43, the two major gap junction connexins (Cxs) in astrocytes, were increased in the striatum of 6-OHDA rats, suggesting that Cx30 levels may be associated with the disease process [14]. Based on the elevated Cx30 immunoreactivity around the vessels in 6-OHDA-treated mice, the authors hypothesised that Cx30 contributes to neurometabolic coupling via its channel-mediated energy transportation [14]. Recently, however, non-channel functions of Cx30 have been investigated [15], and it has been shown that Cx30 can alter astrocyte morphology and modulate their functions, such as synaptic transmission [16]. Thus, the precise roles of Cx30 in DA neurodegeneration remain to be established, especially in terms of non-channel-mediated functions.

We therefore aimed to determine the roles of Cx30 in the pathomechanisms of PD using a 1-methyl-4-phenyl-1,2,3,6-tetrahydropyridine (MPTP) PD model in Cx30 knockout (KO) mice. The goals of this study were to clarify (i) whether the distribution or levels of Cx30 and Cx43 in astrocytes of the nigrostriatal system are altered in response to MPTP, (ii) whether Cx30-deficient mice are sensitive to MPTP toxicity, and (iii) whether the lack of Cx30 induces astrocyte modulation, particularly A1 and A2 astrocytes, under conditions of MPTP toxicity.

## Methods

### Ethical statement

The experimental procedures were designed to minimise the number of animals used as well as animal suffering. All animal experiments were carried out according to the guidelines for the proper conduct of animal experiments published by the Science Council of Japan, and ethical approval for the study was granted by the Animal Care and Use Committee of Kyushu University (#No. A29-179). The Animal Research: Reporting of In Vivo Experiments (ARRIVE) guidelines for animal research were followed.

### Animals

Male heterozygous Cx30 KO mice were obtained from the European Mouse Mutant Archive [17]. Their spermatozoa were used to fertilise C57BL/6 oocytes in vitro. Heterozygous mice were interbred to obtain homozygous Cx30 KO and wild-type (WT) mice. Homozygous non-mutant mice were used as WT controls to ensure the control of the genetic background. All mice were provided food and water ad libitum and kept under a 12-h light/dark cycle in a specific pathogen-free room at the Biomedical Research Laboratory Station for Collaborative Research I of Kyushu University.

### MPTP treatment

Eight-week-old male WT and Cx30 KO mice were intraperitoneally injected with 20 mg/kg of free base MPTP-HCl (Tokyo Chemical Industry, Tokyo, Japan) four times at 2 h intervals, and were sacrificed on days 1 and 7 after the last injection.

### Gene expression microarrays

Total RNA was isolated from the striatum of each animal on day 1 after the last MPTP injection using TRIzol reagent (Thermo Fisher Scientific, Waltham, MA, USA) and purified using the SV Total RNA Isolation System (Promega Corporation, Madison, WI, USA). Total RNA (50 ng) was amplified and labelled with amplification and labelling kits (Agilent Technologies, Santa Clara, CA, USA) and then hybridised to a 60K Agilent 60-mer oligomicroarray (Agilent Technologies). The hybridised microarray slides were scanned using an Agilent Scanner, and the relative hybridisation intensities and background hybridisation values were calculated using Agilent Feature Extraction software (9.5.1.1). Gene set enrichment analysis (GSEA) (www.broadinstitute.org/gsea) was performed to determine the enrichment score (ES), which indicates the degree to which each gene set is overrepresented at the top or bottom of a ranked list of genes. The false discovery rate (FDR) is the estimated probability of an ES representing a false-positive finding. An ES with a normalised *p* value less than 0.05 by an empirical phenotype-based permutation test and an FDR less than 0.25 was considered to be significant. We also calculated *Z*-scores and ratios from the normalised signal intensities of each probe for comparison. *Z*-scores are the number of standard deviations from the mean of log-scaled signal intensities. Ratios are non-log-scaled fold changes in signal intensities. We established criteria of *Z*-score ≥ 2.0 and ratio ≥ 1.5 for upregulated genes and *Z*-score ≤ − 2.0 and ratio ≤ 0.66 for downregulated genes. To determine significantly overrepresented categories of KEGG pathways, we used the tools and datasets provided at the Database for Annotation, Visualisation and Integrated Discovery (DAVID) (http://david.abcc.ncifcrf.gov/home.jsp). The raw data from this study have been submitted to the Gene Expression Omnibus (accession number: GSE113693).

### Immunohistochemistry

Mice were euthanised and transcardially perfused with 4% paraformaldehyde. The brains were removed, fixed in 4% paraformaldehyde overnight, cryopreserved in 30% sucrose in phosphate-buffered saline (PBS), and stored at − 80 °C until analysis. For fluorescent immunostaining, 40-μm-thick sections were cut and incubated overnight at 4 °C with the primary antibodies against Cx30, Cx43, glial fibrillary acidic protein (GFAP), and ionised calcium-binding adapter molecule 1 (Iba1) (Additional file 1: Table S1) and then with an Alexa Fluor 488- or 594-conjugated secondary antibody for 1 h. The sections were mounted in mounting medium containing 4′,6-diamidino-2-phenylindole (DAPI) (Vector Laboratories, Burlingame, CA, USA) and visualised by confocal laser microscopy (Nikon A1, Nikon, Tokyo, Japan). For colorimetric immunostaining, the sections were incubated with primary antibodies against tyrosine hydroxylase (TH), dopamine transporter (DAT), GFAP, and Iba1 (Additional file 1: Table S1) followed by a rabbit or mouse Vectastain Elite ABC HRP kit (Vector Laboratories) and ImmPACT DAB Peroxidase Substrate (Vector Laboratories). We used Nikon NIS-Elements software (Nikon) to calculate the number of Iba1-positive cells, Cx30 dots, and Cx43 dots in the unilateral striata according to the previously reported procedures [18, 19]. The values from the three sections for each animal were averaged.

### In situ hybridisation of *S100a10* and *Gdnf*

Mice were euthanised and perfused as described above. The brains were removed, fixed with G-Fix (Genostaff, Tokyo, Japan), and embedded in paraffin on a CT-Pro20 system (Genostaff) using G-Nox (Genostaff), which is a less toxic organic solvent than xylene. The brains were cut into 8-μm-thick sections and stained as follows with an in situ hybridisation (ISH) Reagent Kit (Genostaff) according to the manufacturer’s instructions. The tissue sections were deparaffinised with G-Nox and rehydrated through a graded ethanol series to PBS. The sections were fixed with 10% formalin in PBS for 30 min at 37 °C, washed with distilled water, placed in 0.2 N HCl for 10 min at 37 °C, washed in PBS, treated with 4 μg/ml proteinase K (Wako Pure Chemical Industries, Osaka, Japan) in PBS for 10 min at 37 °C, and washed again in PBS. The sections were then placed in a Coplin jar containing 1× G-Wash (Genostaff; equivalent to 1× saline sodium citrate buffer). Hybridisation was performed by incubation with probes for *S100a10* and *Gdnf* (Additional file 1: Table S2) at 250 ng/ml in G-Hybo-L (Genostaff) for 16 h at 60 °C. The sections were then washed in 1× G-Wash for 10 min at 60 °C and incubated in 50% formamide in 1× G-Wash for 10 min at 60 °C. The sections were washed twice in 1× G-Wash for 10 min at 60 °C, twice in 0.1× G-Wash for 10 min at 60 °C, and twice in 0.1% Tween-20 in Tris-buffered saline (TBST) at room temperature. The sections were then incubated with 1× G-Block (Genostaff) for 15 min at room temperature and with alkaline phosphatase-coupled anti-digoxigenin (Roche Diagnostics, Mannheim, Germany) diluted 1:2000 with 1× 50 G-Block (Genostaff) in TBST for 1 h at room temperature. The sections were washed twice in TBST and then incubated in 100 mM NaCl, 50 mM MgCl_2_, 0.1% Tween-20, 100 mM Tris-HCl, and pH 9.5. Colour development was performed by incubation with nitro blue tetrazolium and 5-bromo-4-chloro-3′-indolyphosphate solution (Sigma-Aldrich, St. Louis, MO, USA) overnight followed by washing in PBS.

Following ISH, S100β protein was detected by IHC. The sections were incubated with 0.3% H_2_O_2_ in PBS for 30 min to block endogenous peroxidase and then incubated with G-Block (Genostaff) and Avidin/Biotin Blocking reagent (Vector Laboratories). The sections were then incubated successively with anti-S100β antibody (Abcam) at 4 °C overnight, biotin-conjugated goat anti-rabbit antibody (Dako, Santa Clara, CA, USA) for 30 min at room temperature, and peroxidase-conjugated streptavidin (Nichirei, Tokyo, Japan) for 5 min. Peroxidase activity was visualised by incubation with diaminobenzidine, and the sections were mounted with G-Mount (Genostaff). Images of the bilateral striata were captured using a Leica DM2500 microscope with a × 40 objective (Leica Microsystems, Wetzlar, Germany). Finally, the number of cells double positive for S100β protein and either *Gdnf* or *S100a10* mRNA was counted. The primer sequences specific for *Gdnf* and *S100a10* are listed in Additional file 1: Table S2.

### Stereology

The total numbers of TH-, GFAP-, and Iba1-positive cells in unilateral SNcs were measured stereologically using an optical fractionator method as previously described [20–22]. Every fourth section through the SNc was analysed using Stereo Investigator software (Stereo Investigator 10.0; MicroBrightField, Williston, VT, USA). Immunolabelled cells were counted by the optical fractionator method (× 40 objective; counting frame, 100 × 100 μm; sampling grid, 200 × 200 μm; counting frame thickness, 10 μm). The coefficient of error (Gundersen, *m* = 1) for cell count estimation was less than 0.15 for each animal.

### Western blotting of Cx30, Cx43, and GFAP in striatum specimens

Striata were rapidly isolated and homogenised on ice in a radioimmunoprecipitation buffer containing a protease inhibitor cocktail, 0.5% sodium dodecyl sulphate (Nacalai Tesque, Kyoto, Japan), and PhosSTOP phosphatase inhibitor cocktail (Roche Diagnostics). Lysates were incubated on ice for 30 min, sonicated, and then centrifuged at 4 °C for 10 min at 10,000×*g*. Supernatants were removed, and protein concentrations were determined using a BCA protein assay kit (Thermo Fisher Scientific). The samples were mixed with Laemmli buffer with (Cx43 and GFAP) or without (Cx30) heating to 95 °C for 5 min. Proteins were separated by polyacrylamide gel electrophoresis (12% for Cx30 and 7.5–15% gradient for Cx43 and GFAP) and blotted onto polyvinyl difluoride membranes. The membranes were blocked with 3% nonfat milk (for Cx30) or Blocking One (Nacalai Tesque; for Cx43 and GFAP) and then incubated with anti-Cx30 (1: 500; Invitrogen), anti-Cx43 (1: 10,000; Abcam), or anti-GFAP antibodies (1: 2000; STEMCELL Technologies) overnight at 4 °C or with anti-β-actin antibody (1: 20,000; Sigma-Aldrich) for 1 h at room temperature. The membranes were then incubated with a horseradish peroxidase-conjugated secondary antibody for 1 h at room temperature. The immunoreactive protein bands were visualised by enhanced chemiluminescence (ECL Prime, GE Healthcare Bio-Sciences, Uppsala, Sweden). Band intensities were measured using the ChemiDoc™ XRS system (Bio-Rad Laboratories) and normalised to β-actin levels.

### Analysis of TH- and DAT-positive fibre density

The density of TH- and DAT-positive fibres in the striatum was assessed in the sections between Bregma + 0.62 and − 0.10 mm, as previously reported [23]. Every fourth section was immunolabelled, to give a total of six sections analysed per animal. Four to six images per section were captured using an Olympus B51 microscope with a × 100 objective (Olympus Corp., Tokyo, Japan). The optical density was measured using ImageJ software (National Institutes of Health, Bethesda, MD, USA), followed by background subtraction of the corpus callosum.

### RNA extraction and quantitative reverse transcription polymerase chain reaction (RT-PCR) of *Cx30*, *Cx43*, *S100a10*, and *Gdnf* mRNA in the striatum

Total RNA was isolated from the striata using an RNA purification kit (Qiagen, Hilden, Germany), and cDNA was synthesised using ReverTra Ace qPCR RT Master Mix with gDNA Remover (Toyobo, Osaka, Japan). Quantitative PCR was performed using an Applied Biosystems 7500 Real-Time PCR System (Thermo Fisher Scientific) with TaqMan Gene Expression Master Mix (Thermo Fisher Scientific) and TaqMan Gene Expression Assays (*Cx30*, Mm00433661_s1; *Cx43*, Mm01179639_s1; *S100a10*, Mm00501457_m1; *Gdnf*, Mm01285715_m1; Thermo Fisher Scientific). Glyceraldehyde 3-phosphate dehydrogenase (*Gapdh*, Mm99999915_g1) was amplified as an internal control. The ∆∆CT efficiency correction method was used to calculate relative mRNA levels.

### Measurement of GDNF protein level in the striatum by enzyme-linked immunosorbent assay (ELISA)

The striata were rapidly isolated and homogenised on ice in a radioimmunoprecipitation buffer containing a protease inhibitor cocktail (Nacalai Tesque). Lysates were incubated on ice for 30 min, sonicated, and centrifuged at 4 °C for 10 min at 10,000×*g*. Supernatants were removed, and protein concentrations were determined using a BCA protein assay kit (Thermo Fisher Scientific). GDNF was measured using a specific ELISA kit (Affymetrix, Santa Clara, CA, USA).

### Measurement of dopamine, 3,4-dihydroxyphenylacetic acid (DOPAC), and homovanillic acid (HVA) levels by high-performance liquid chromatography with electrochemical detection (HPLC-ECD)

The striata were weighed and stored at − 80 °C until analysis. Frozen striata were sonicated in 300 μl of ice-cold 0.1 N perchloric acid and 0.2 mM sodium bisulphite solution and centrifuged at 4 °C for 20 min at 20,000×*g*. The supernatant was filtered and analysed by HPLC-ECD. Aliquots (10 μl) of each sample were separated on a C18 reverse phase column (Capcell Pak column, 150 × 4.6 mm, Shiseido, Tokyo, Japan) and detected with an ECD system consisting of a Coulochem III controller (ESA, Inc., Chelmsford, MA, USA) fitted with a guard cell (M5020) and an analytical cell set (M5011). The guard cell was set at 450 mV, electrode 1 at 50 mV, and electrode 2 at 400 mV. The mobile phase was phosphate buffer containing heptanesulfonic acid, and the flow rate was 0.8 ml/min.

### Measurement of striatal 1 methyl-4-phenylpyridinium (MPP^+^) levels

Mice were injected once with MPTP and euthanised 2 h later. The brains were removed, and the striata were weighed, placed in 100 μl of tissue buffer (0.1 M phosphate-citric acid buffer, pH 2.5, containing 20% methanol), and centrifuged for 1 min at 13,000×*g*. Aliquots (2 μl) of each supernatant sample were applied to a liquid chromatography-tandem quadrupole mass spectrometer (LC-MS/MS) with electrospray ionisation in positive mode. LC was performed on a Waters UltraPerformance LC system (Waters Corporation, Milford, MA, USA) with a Waters BEH C18 column (2.1 mm × 100 mm) maintained at 40 °C. The mobile phase consisted of 0.1% formic acid in water (solvent A) and acetonitrile (solvent B). Separation was performed starting at 5% B increasing to 95% B over 5 min. The flow rate was 0.3 ml/min.

### Statistical analysis

All values are expressed as the means ± standard error of mean (SEM). Differences were analysed using two-way analysis of variance (ANOVA) for more than two groups, Dunnett’s *t* test for two groups, and Pearson’s chi-square test for comparison of mortality rates. When ANOVA showed significant differences, pairwise comparisons were assessed using the Tukey–Kramer post hoc test. The null hypothesis was rejected at the 0.05 level.

## Results

### MPTP upregulates Cx30 expression

**Fig. 1:**
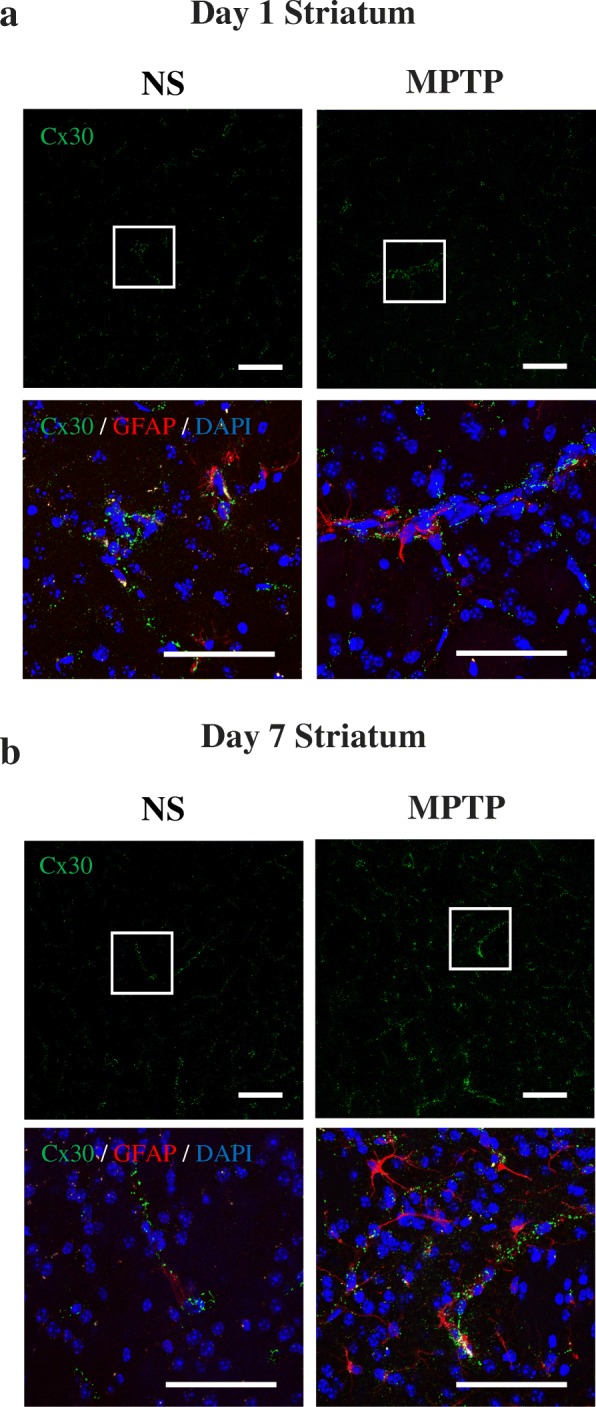
Cx30 immunostaining in the striatum. **a**, **b** Upper panels: Cx30 immunostaining in the striatum in WT mice 1 day (**a**) and 7 days (**b**) after injection of normal saline (NS) or MPTP. Lower panels: Enlarged images showing the areas marked with a white box in the upper panels. The sections were triple-stained for Cx30 (green), GFAP (red), and DAPI (blue, nuclei). Scale bars: upper panels, 100 μm; lower panels, 50 μm

**Fig. 2:**
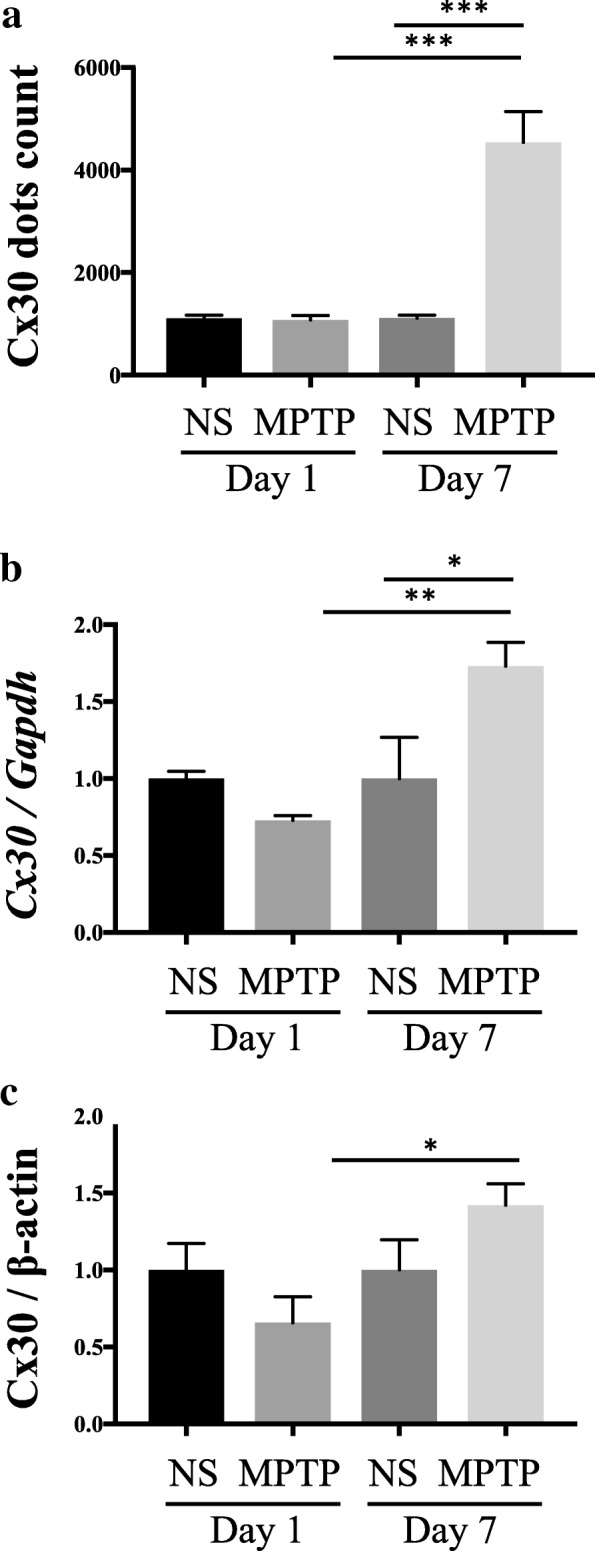
Effects of MPTP on Cx30 levels in the striatum of WT mice on days 1 and 7 after injection of normal saline (NS) or MPTP. **a** Number of Cx30-immunoreactive dots counted by Nikon NIS-Element software. **b** qRT-PCR analysis of *Cx30* mRNA levels normalised to *Gapdh* mRNA. **c** Cx30 protein levels measured by Western blotting and normalised to β-actin levels. Data are the mean ± SEM of *n* = 4–5 mice per group. *p < 0.05, **p < 0.01, and ***p < 0.001 by two-way ANOVA followed by the Tukey–Kramer post hoc test. Blot images are presented in Additional file 1: Figure S1

We first examined the changes in Cx30 expression in the sections of the striatum and SNc from WT mice on treated with normal saline (NS, control) or MPTP for 1 or 7 days. Immunofluorescence staining revealed Cx30 expression as small foci or dots (Fig. 1). The number of Cx30 dots was significantly increased between days 1 and 7 in MPTP-treated, but not NS-treated, mice (*p* < 0.0001, Fig. 2a), suggesting that MPTP treatment increased the expression of Cx30. This finding was supported by qRT-PCR and Western blot analysis which showed a significant increase in *Cx30* mRNA levels (*p* = 0.0044, Fig. 2b) and Cx30 protein levels (*p* = 0.0269, Fig. 2c) in the striata of WT mice between days 1 and 7 after MPTP treatment (Additional file 1: Figures S1 and S2).

MPTP had no effects on the abundance of immunoreactive Cx30 dot counts or levels of *Cx30* mRNA and protein in the striatum compared with NS on 1 day after treatment. However, on day 7, the striata from MPTP-treated mice contained significantly more Cx30 dots (*p* < 0.0001, Fig. 2a) and significantly higher *Cx30* mRNA levels (*p* = 0.0277, Fig. 2b) than did the striata from NS-treated mice. In contrast, Cx30 protein levels were insignificantly higher in the MPTP-treated mice than NS-treated mice on day 7 post-treatment (*p* = 0.3302, Fig. 2c).

By immunofluorescence microscopy, small immunoreactive Cx30 dots were detectable along the striatal blood vessels of NS-treated mice, and their density in hypertrophic astrocytes was markedly increased 7 days after MPTP administration (Fig. 1a, b, upper panels). These findings are consistent with a previous report showing that Cx30 localisation is strongly increased around the striatal vessels following treatment with 6-OHDA [14]. Cx30 dots were also observed outside the perivascular areas of MPTP-treated mice, predominantly along astrocyte processes and occasionally in astrocyte cell bodies (Fig. 1a, b, lower panels). In the SNc, Cx30 expression was strongly upregulated by MPTP treatment and was present in both astrocyte processes and cell bodies (Additional file 1: Figure S3). To confirm the specificity of this analysis, we performed anti-Cx30 immunostaining of NS- and MPTP-treated striata from Cx30 KO mice, and we detected no Cx30 immunoreactivity (Additional file 1: Figure S4).

### MPTP upregulates Cx43 expression

**Fig. 3:**
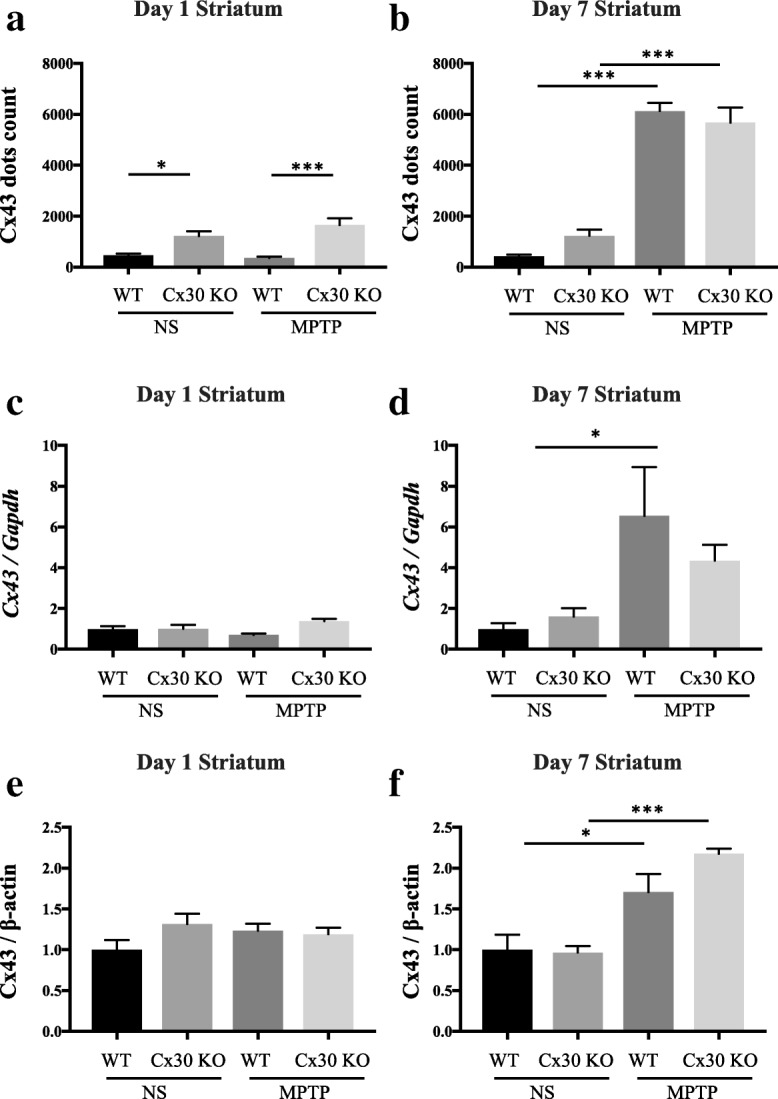
Effects of MPTP on Cx43 expression in the striatum of WT and Cx30 KO mice on days 1 and 7 after the treatment with normal saline (NS) or MPTP. **a**, **b** Number of Cx43-immunoreactive dots counted by Nikon NIS-Element software. **c**, **d**, qRT-PCR analysis of *Cx30* mRNA levels normalised to *Gapdh* mRNA. **e**, **f** Cx30 protein levels measured by Western blotting and normalised to β-actin levels. Analyses were performed on day 1 (**a**, **c**, **e**) and day 7 (**b**, **d**, **f**) after treatment. Data are presented as the mean ± SEM of *n* = 4–5 mice per group. *p < 0.05 and ***p < 0.001 by two-way ANOVA followed by the Tukey–Kramer post hoc test. Blot images are presented in Additional file 1: Figure S2

To determine whether Cx30 expression influences the expression of a second astrocyte gap junction protein, Cx43, we performed a similar analysis of Cx43 expression in the striatum and SNc of WT and Cx30 KO mice. As was observed for Cx30, Cx43 was detectable as fluorescent dots by IHC (Additional file 1: Figure S5). In both WT and Cx30 KO mice, the number of Cx43 dot counts did not change between days 1 and 7 in NS-treated mice but increased significantly between days 1 and 7 after MPTP treatment (*p* < 0.0001 for WT and Cx30 KO mice, Additional file 1: Figure S6a, b). As a result, Cx43 dot counts were comparable in NS- and MPTP-treated WT mice on day 1, but they were significantly higher in MPTP-treated than NS-treated WT mice on day 7 (*p* < 0.0001, Additional file 1: Figure S6a, b). The same trend was observed for Cx30 KO mice (*p* < 0.0001, Additional file 1: Figure S6a, b). Moreover, deletion of Cx30 caused a significant increase in the number of Cx43 dots in the striatum on day 1 after NS and MPTP treatment (*p* = 0.0310 and *p* = 0.0007, respectively, Fig. 3a) but not on day 7 (Fig. 3b).

RT-PCR analysis showed that *Cx43* mRNA levels in the striatum were unchanged by NS treatment but were significantly increased by MPTP treatment between days 1 and 7 in both WT and Cx30 KO mice (*p* = 0.0322 and *p* = 0.0038, respectively, Additional file 1: Figure S6c, d). The levels of *Cx43* mRNA did not differ significantly between NS- and MPTP-treated mice on day 1 after treatment, but they were significantly different after 7 days of treatment, with MPTP treatment inducing a marked increase in both WT and Cx30 KO mice (*p* = 0.0304 and *p* = 0.0044, respectively, Additional file 1: Figure S6c, d). Moreover, deletion of Cx30 caused no significant change in *Cx43* mRNA levels in the striatum on days 1 and 7 after NS and MPTP treatment (Fig. 3c, d).

Cx43 protein levels did not change between days 1 and 7 after NS treatment in WT or Cx30 KO mice (Additional file 1: Figures S6e, S6f, S7, S8), whereas MPTP treatment significantly increased Cx43 protein expression between day 1 in Cx30 KO mice (*p* < 0.0001) but not in WT mice (*p* = 0.2795; Additional file 1: Figures S6e, f, S7, S8). As a result, Cx43 protein levels were comparable in NS- and MPTP-treated WT mice on day 1, but they were significantly higher in MPTP-treated than NS-treated WT mice on day 7 (*p* = 0.043, Additional file 1: Figure S6e, f). The same trend was observed for Cx30 KO mice (*p* < 0.0001, Additional file 1: Figure S6e, f). Moreover, deletion of Cx30 caused no significant change in Cx43 protein levels in the striatum on days 1 and 7 after NS and MPTP treatment (Fig. 3e, f).

**Fig. 4:**
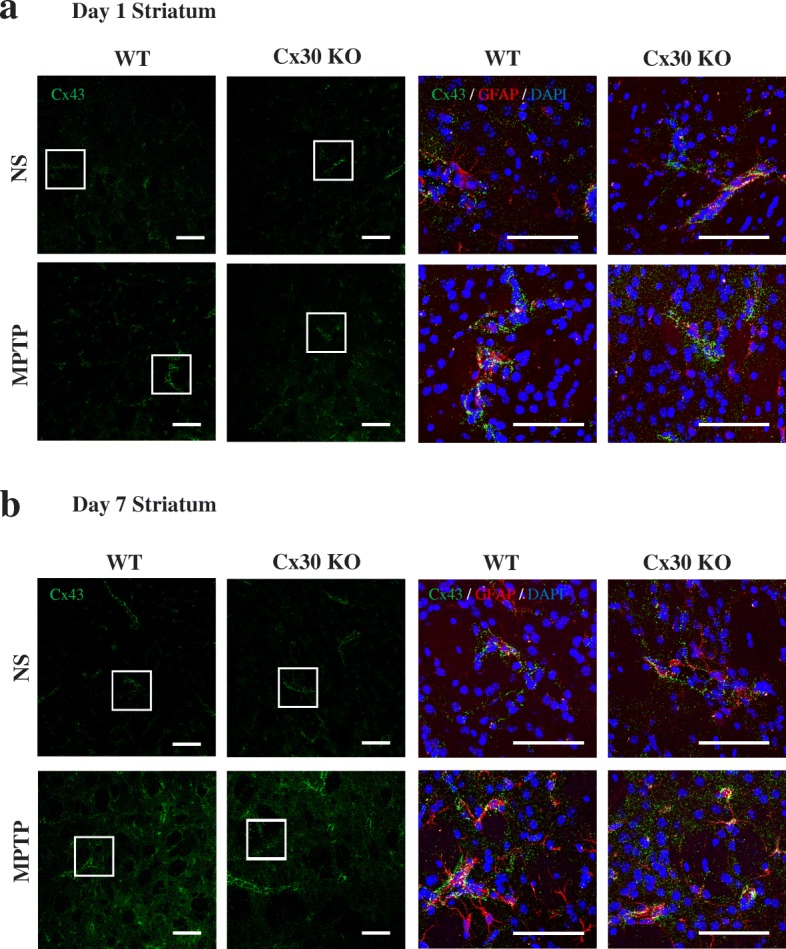
Cx43 immunostaining in the striatum. Left two columns: Cx43 immunostaining in the striatum of WT and Cx30 KO mice on day 1 (**a**) and 7 (**b**) after injection of normal saline (NS) or MPTP. Right two columns: Enlarged images showing the areas marked with a white box in the left columns. The sections were triple-stained for Cx43 (green), GFAP (red), and DAPI (blue, nuclei). Scale bars: left columns, 100 μm; right columns, 50 μm

On day 1 after NS or MPTP treatment, Cx43 dots were predominantly observed in astrocyte processes along the blood vessels in the striatum of WT and Cx30 KO mice (Fig. 4a); in contrast, Cx43 dots were also present beyond the perivascular areas by day 7 (Fig. 4b), which was similar to the pattern of Cx30 dots 7 days after MPTP treatment. In the SNc, the pattern of Cx43 staining was similar to that of Cx30. Cx43 dots were mainly detected in astrocyte processes along the vessels on day 1 after NS and MPTP treatment of both WT and Cx30 KO mice, and this was upregulated on day 7 after MPTP treatment (Additional file 1: Figure S5). These results suggest that Cx30 deficiency modestly increases the basal Cx43 expression and MPTP-upregulated Cx43 expression on day 1 after administration. This may reflect a partial compensation for the loss of Cx30, as observed in Cx43-deficient mice, which showed a twofold increase in Cx30 expression [24]. However, Cx30 deficiency did not alter either the levels or distribution of Cx43 on day 7 after MPTP treatment.

### Cx30 deficiency does not change MPP^+^ production or DAT expression

MPTP is converted by astrocytes to the neurotoxic molecule MPP^+^, which is then taken up into DA neurons via DAT. We used LC-MS/MS to measure MPP^+^ concentrations in the striatum of WT and Cx30 KO mice at 2 h after a single MPTP injection (peak MPP^+^ brain concentrations are observed at 1 h after injection [25]). We found no significant difference in the concentration of MPP^+^ in WT and Cx30 KO mice (Additional file 1: Figure S9), suggesting that Cx30 deficiency does not affect astrocyte MPTP metabolism. In addition, there was no significant difference between WT and Cx30 KO mice in DAT intensity (Additional file 1: Figure S10). Taken together, these results suggest that MPP^+^ uptake into DA neurons is comparable in WT and Cx30 KO mice.

### Cx30 deficiency accelerates MPTP-induced loss of DA neurons

**Fig. 5:**
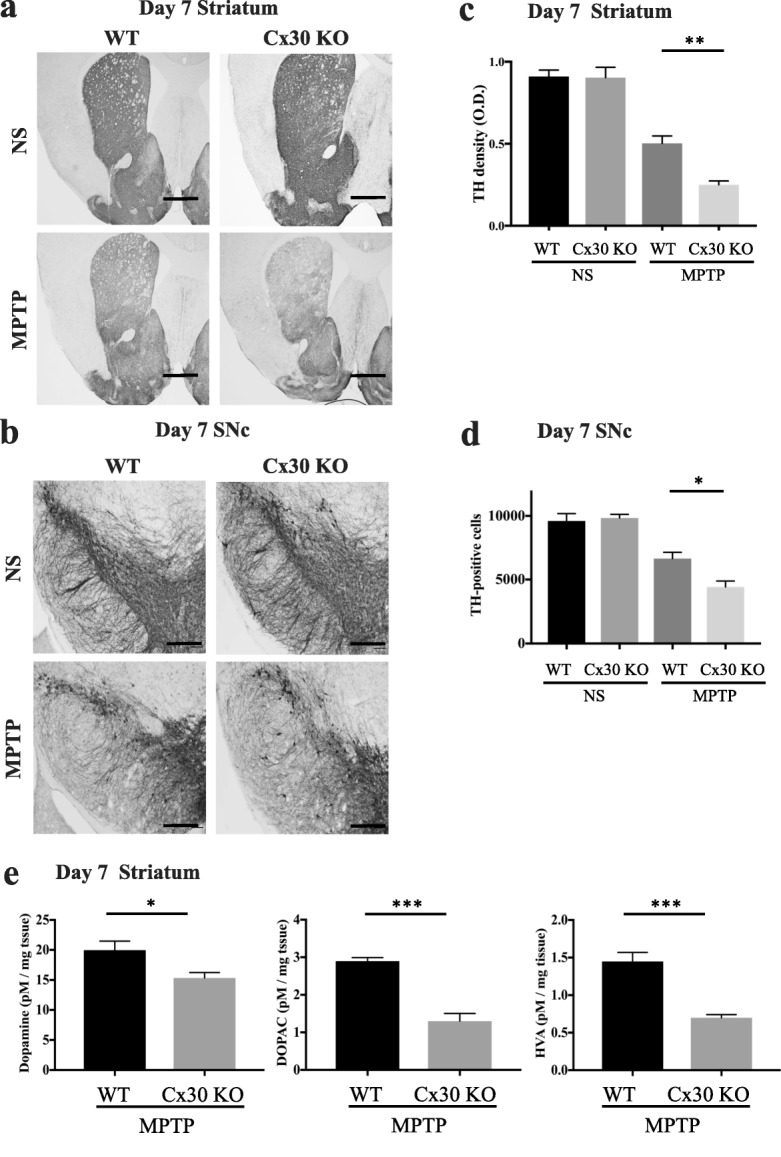
Acceleration of MPTP-induced nigrostriatal damage by Cx30 deficiency. WT and Cx30 KO mice were injected with normal saline (NS) or MPTP, and mice were analysed 7 days later. **a**, **b** TH immunostaining of the striatum (**a**) and the SNc (**b**) in scale bars **a** 50 μm and **b** 100 μm. **c**, **d** TH-positive fibre density in the striatum (**c**) and number of TH-positive cells in the SNc (**d**). **e** Concentrations of dopamine and its metabolites DOPAC and HVA in the striatum. The experiment to examine the MPTP-induced acceleration of DA neuron death was repeated three times, and essentially, the same results were obtained. Data are presented as the mean ± SEM of *n* = 4 mice per group. *p < 0.05, **p < 0.01, and ***p < 0.001 by two-way ANOVA followed by the Tukey–Kramer post hoc test (**c**, **d**) or Dunnett’s *t* test (**e**)

Next, we examined the susceptibility of WT and Cx30 KO mice to MPTP neurotoxicity. MPTP caused severe acute toxicity, including immobility and, occasionally, death, in both the WT and Cx30 KO mice, as previously reported [26, 27]. The mortality rates on day 1 after MPTP treatment were higher in Cx30 KO mice than in WT mice, albeit not significantly (38% (19/50) vs. 20% (10/49), *p* = 0.0545). To evaluate DA neuron damage, we quantified TH-positive cells and dopamine levels at 7 days after MPTP injection. This time point was selected because TH expression and dopamine levels in the striatum of MPTP-treated mice have been reported to gradually decrease after 7 days, and the number of TH-positive cells in the SNc reaches a nadir 2 days after MPTP injection and then stabilises for at least 7 days thereafter [28]. We found that TH-positive fibre densities in the striatum and TH-positive cell numbers in the SNc were significantly decreased in Cx30 KO mice compared with WT mice after MPTP treatment (*p* = 0.0018 and *p* = 0.0246, respectively; Fig. 5a–d). Similarly, striatal levels of dopamine and its metabolites DOPAC and HVA were significantly lower in Cx30 KO mice than in WT mice after MPTP treatment (*p* = 0.0372, *p* < 0.0001, and *p* = 0.0007, respectively; Fig. 5e). Taken together, these findings indicate that Cx30 KO mice display hypersensitivity to MPTP.

### Cx30 deficiency reduces A2 and pan-reactive astrocyte responses 1 day after MPTP treatment

**Fig. 6:**
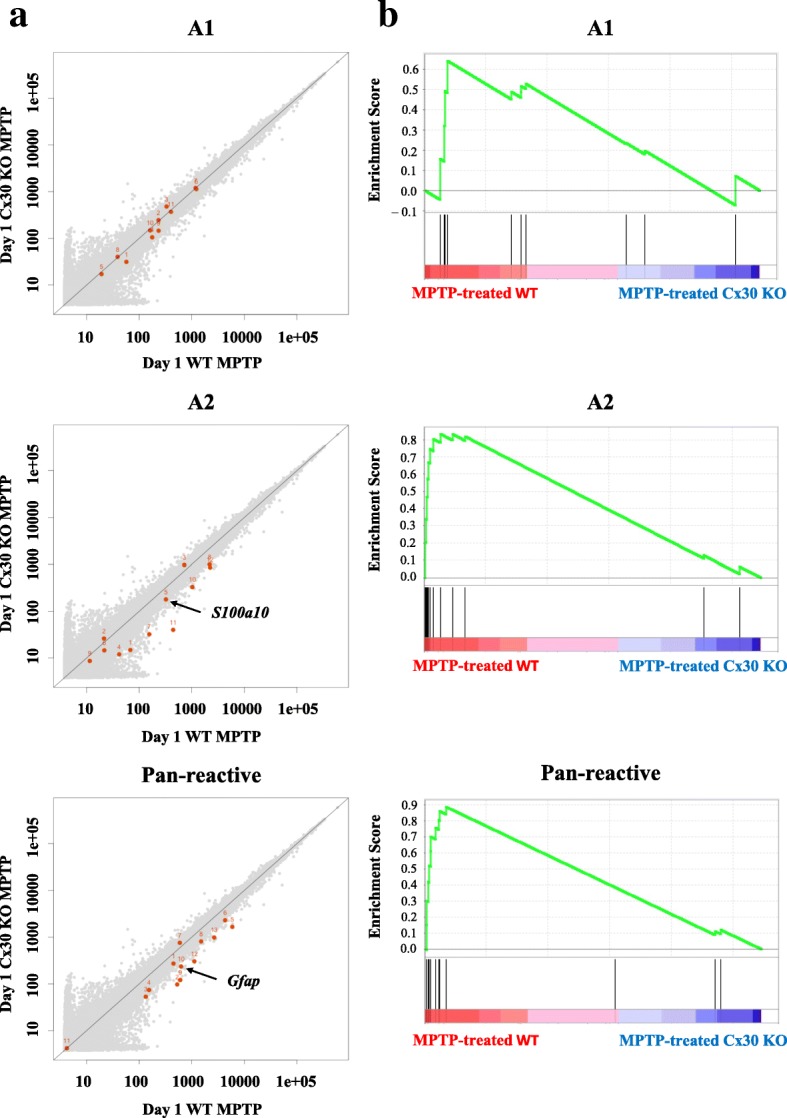
Microarray gene expression analysis of A1, A2, and pan-reactive astrocyte gene sets at day 1 after MPTP treatment. **a** Scatter plots. Black arrows show the genes that were validated by RT-PCR. The *X*- and *Y*-axis values are the log10 scale-normalised signals. **b** Enrichment plots (green curve) show the running sum of the enrichment score (ES) for each gene set. The score at the peak of the plots is the ES for each gene set. The black bars show where the members of the gene set appear in the ranked list of genes. A predominance of black bars to the left or right side indicates that most genes are upregulated in MPTP-treated WT mice or MPTP-treated Cx30 KO mice, respectively

Because Cx30 deficiency in astrocytes may alter their response to brain insults, we analysed the changes in gene expression associated with A1, A2, and pan-reactive astrocyte responses in the striatum on day 1 after NS or MPTP injection. Comparative microarray analyses showed that MPTP-induced gene expression changes in A1 astrocytes were similar in WT and Cx30 KO mice, whereas MPTP-induced upregulation of A2 and pan-reactive gene expression observed in WT mice was markedly attenuated in Cx30 KO mice (Fig. 6a; Additional file 1: Figure S11 and Table S3). Consistent with this, GSEA showed that the A2 and pan-reactive gene sets were significantly enriched in WT compared with Cx30 KO mice (A1: ES = 0.6435, normalised *p* = 0.1523, FDR = 0.1836; A2: ES = 0.8335, normalised *p* < 0.0001, FDR = 0.0076; pan-reactive: ES = 0.8886, normalised *p* < 0.0001, FDR < 0.0001; Fig. 6b). These data indicate that Cx30 is required for optimal induction of neuroprotective A2, as well as pan-reactive astrocyte responses against MPTP toxicity.

### Cx30 deficiency reduces MPTP-induced *S100a10* upregulation and decreases constitutive expression of *Gdnf* in the striatum

**Fig. 7:**
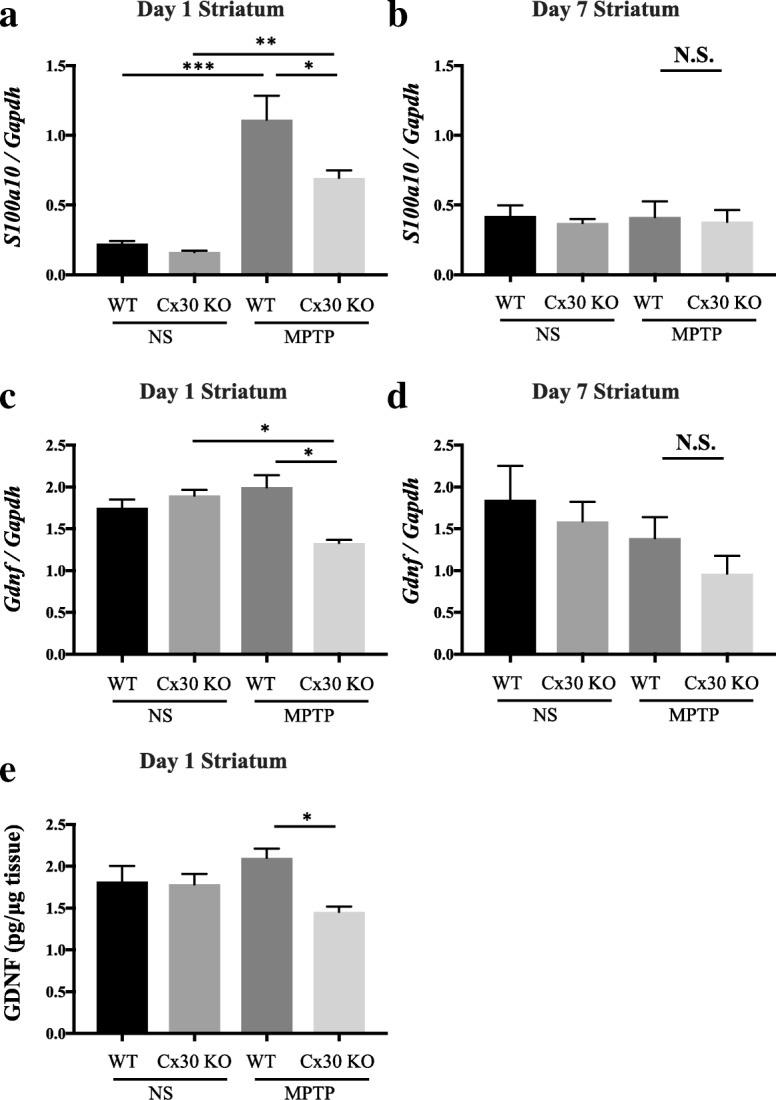
Alteration of *S100a10* and *Gdnf* expression in the striatum by Cx30 deficiency. WT and Cx30 KO mice were injected with normal saline (NS) or MPTP, and mice were analysed 1 and 7 days later. **a**, **b** RT-PCR analysis of *S100a10* mRNA levels normalised to *Gapdh* mRNA. **c**, **d** RT-PCR analysis of *Gdnf* mRNA levels normalised to *Gapdh* mRNA. **e** ELISA analysis of GDNF protein levels. Analyses were performed on days 1 (**a**, **c**, **e**) and 7 (**b**, **d**) after treatment. Data are expressed as the mean ± SEM of *n* = 4–5 mice per group. N.S., not significant; *p < 0.05, **p < 0.01, and ***p < 0.001 by two-way ANOVA followed by the Tukey–Kramer post hoc test

One of the A2 astrocyte genes that showed a reduced response to MPTP in Cx30 KO mice compared with WT mice was *S100a10* (*Z*-score = − 3.9153, ratio = 0.4566). We selected this for further analysis because S100A10 plays important roles in intracellular trafficking and cell migration, thereby contributing to neuroprotection [29, 30]. RT-PCR analyses of striatal tissues 1 day after treatment demonstrated that MPTP significantly increased *S100a10* mRNA expression compared with NS treatment in both WT and Cx30 KO mice (*p* < 0.0001 and *p* = 0.0063, respectively), but the upregulation in Cx30 KO mice was significantly less than that in WT mice (*p* = 0.0303; Fig. 7a), in agreement with the microarray results. However, these differences were not observed on day 7 after treatment (Fig. 7b).

The microarray analyses revealed that the expression of *Gdnf*, a well-known neurotrophic factor constitutively expressed in the striatum [31], was decreased in Cx30 KO mice compared with WT mice after MPTP treatment (*Z*-score = − 2.3141 and ratio = 0.5661). Using RT-PCR, we confirmed that striatal *Gdnf* expression was significantly decreased in Cx30 KO mice compared with WT mice 1 day after MPTP treatment (*p* = 0.0139), and its expression in Cx30 KO mice was significantly lower on day 1 after MPTP treatment compared with NS treatment (*p* = 0.0275; Fig. 7c). Similar to *S100a10*, *Gdnf* mRNA levels were not significantly different between WT and Cx30 KO mice at 7 days after treatment with NS or MPTP (Fig. 7d). ELISA analysis of GDNF protein in the striatum 1 day after MPTP treatment showed that levels were significantly lower in Cx30 KO mice compared with WT mice (*p* = 0.0192; Fig. 7e), consistent with the RT-PCR results. Collectively, these findings suggest that expression of both *S100a10*, a representative MPTP-upregulated A2 astrocyte response gene, and *Gdnf*, a constitutively expressed neuroprotective factor, is lower in Cx30 KO mice compared with WT mice at 1 day, but not 7 days, after MPTP administration.

**Fig. 8:**
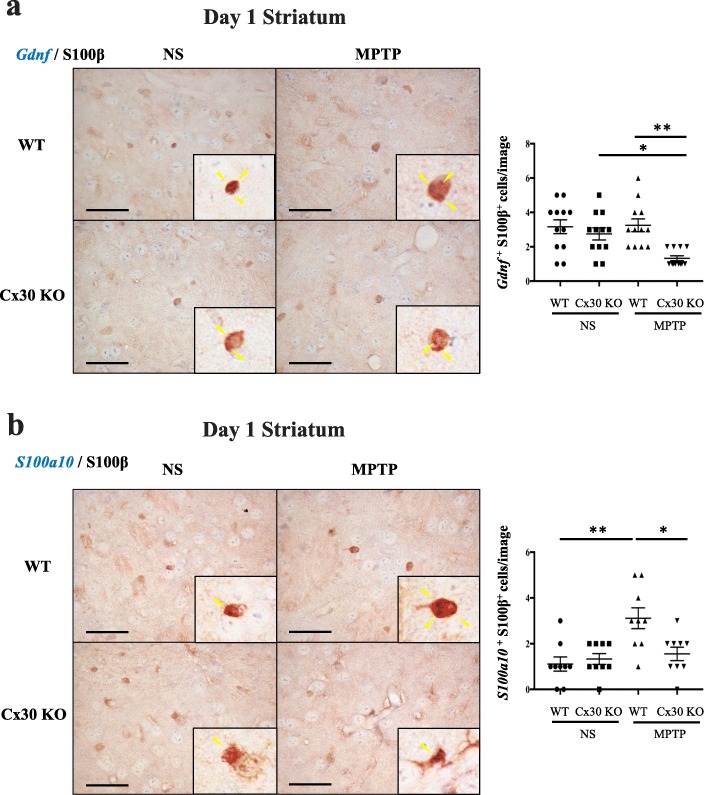
Expression of *Gdnf* and *S100a10* mRNA in S100β-positive astrocytes. Left panels: *Gdnf* (**a**) and *S100a10* (**b**) in situ hybridisation (blue colour) combined with S100β immunohistochemistry (brown colour) in the striatum of WT and Cx30 KO mice 1 day after injection of normal saline (NS) or MPTP. *Gdnf* and *S100a10* mRNA expression (yellow arrowheads) colocalise with S100β-positive astrocytes. Black boxes show enlarged images in each picture. Right panels: Quantification of the number of *Gdnf* and S100β double-positive cells (**a**) and *S100a10* and S100β double-positive cells (**b**). Each point is a data from an individual image. Horizontal bars represent the mean ± SEM of *n* = 12 (**a**) or 9 (**b**) images per group. *p < 0.05 and **p < 0.01 by two-way ANOVA followed by the Tukey–Kramer post hoc test. Scale bars, 10 μm

We performed ISH combined with IHC on the striatal sections to detect cells in which *Gdnf* or *S100a10* mRNA were colocalised with S100β protein. We detected *Gdnf* or *S100a10* mRNA and S100β-positive striatal astrocytes and other cell types that were *Gdnf* or *S100a10* mRNA-positive S100β-negative in both WT and Cx30 KO mice after treatment with NS or MPTP (Fig. 8a, b). MPTP had no effect on the number of *Gdnf* and S100β double-positive cells in WT mice, while it significantly decreased the number in Cx30 KO mice (*p* = 0.0221, Fig. 8a). As a result, the number of *Gdnf* and S100β double-positive cells was significantly lower in Cx30 KO mice compared with WT mice at 1 day after MPTP treatment (*p* = 0.0011, Fig. 8a). The number of *S100a10* and S100β double-positive cells was significantly increased after MPTP treatment in WT mice (*p* = 0.0010, Fig. 8b) but was unchanged in Cx30 KO mice (Fig. 8b). As a result, the number of *S100a10* and S100β double-positive cells was significantly lower in Cx30 KO mice compared with WT mice at 1 day after MPTP treatment (*p* = 0.0121, Fig. 8b).

### Cx30 deficiency inhibits upregulation of GFAP protein after MPTP treatment

**Fig. 9:**
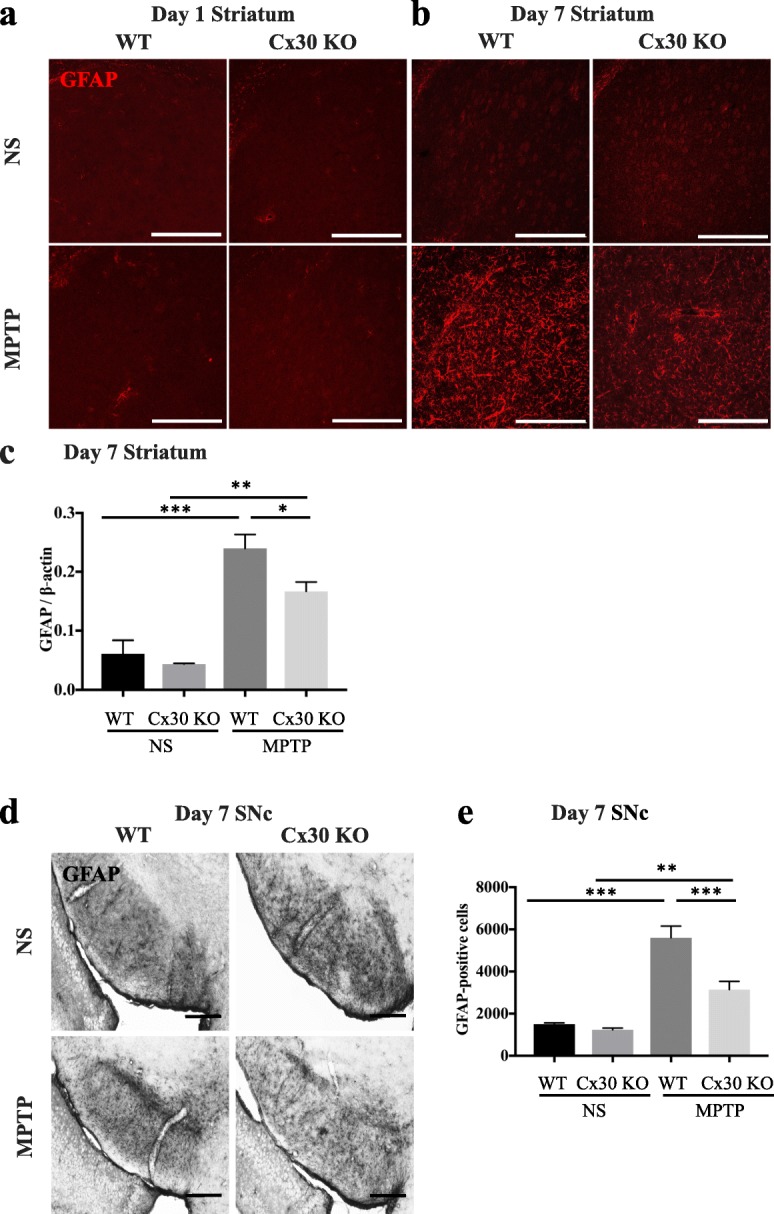
Reduced MPTP-induced GFAP upregulation in Cx30-deficient mice. **a**, **b** GFAP immunostaining of the striatum on day 1 (**a**) and 7 (**b**) after injection of WT and Cx30 KO mice with normal saline (NS) or MPTP. **c** Quantification of Western blot analysis of GFAP protein levels normalised to β-actin levels. **d**, **e** GFAP immunostaining (**d**) and GFAP-positive cell numbers (**e**) in the SNc in WT and Cx30 KO mice 7 days after injection of NS or MPTP. Data are the mean ± SEM of *n* = 3 (**c**) or 4 (**e**) mice. *p < 0.05, **p < 0.01, and***p < 0.001 by two-way ANOVA followed by the Tukey–Kramer post hoc test. Scale bars, 200 μm (**a**, **b**) and 100 μm (**d**). Blot images are presented in Additional file 1: Figure S11

In the microarray analysis of pan-reactive astrocyte genes, *Gfap* expression was upregulated by MPTP to a smaller extent in Cx30 KO mice than in WT mice (*Z*-score = − 6.9796 and ratio = 0.2812). To explore this further, we analysed GFAP protein expression levels in the striatum and SNc by IHC and Western blotting. We detected no effects of MPTP on striatal GFAP expression on day 1 after MPTP treatment in either mouse strain (Fig. 9a). However, on day 7, a robust MPTP-induced increase in GFAP levels was observed in WT and Cx30 KO mice, although the change in Cx30 KO mice was less marked (Fig. 9b), as confirmed by Western blotting (*p* = 0.0346; Fig. 9c and Additional file 1: Figure S12). Stereological counting of GFAP-positive cells in the SNc also revealed significantly fewer cells in the SNc of Cx30 KO mice compared with WT mice on day 7 after MPTP treatment (*p* = 0.0008; Fig. 9d, e). Taken together with the microarray results, these findings indicate that Cx30 deficiency reduces the MPTP-induced upregulation of *Gfap*, a pan-reactive astrocyte gene, at both the mRNA and protein levels.

### Cx30 deficiency does not affect the microglial response to MPTP treatment

**Fig. 10:**
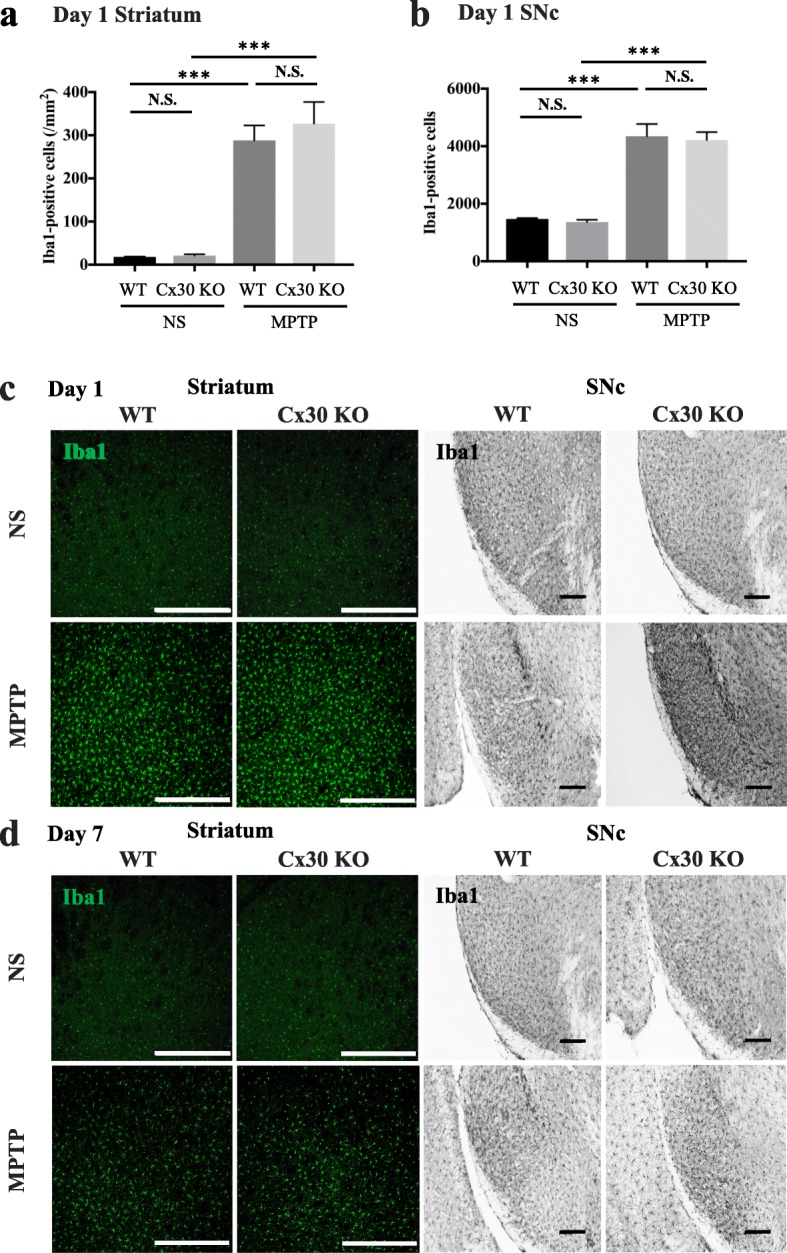
Similar microglial activation in MPTP-treated WT and Cx30 KO. WT and Cx30 KO mice were injected with normal saline (NS) or MPTP and analysed on day 1 or 7 after treatment. **a**, **b** Number of Iba1-positive cells in the striatum (**a**) and SNc (**b**) of WT and Cx30 KO mice on day 1. **c**, **d** Iba1 immunostaining of the striatum and SNc on days 1 (**c**) and 7 (**d**) after treatment. Data are expressed as the mean ± SEM of *n =* 4 mice per group. N.S., not significant; ***p < 0.001 by two-way ANOVA followed by the Tukey–Kramer post hoc test. Scale bars: striatum, 200 μm; SNc, 100 μm

Microglia play a critical role in neuroinflammation in PD; therefore, we examined microglial activation in our PD mouse model [32]. Previous work has shown that microglia are activated to a greater extent on day 1 after MPTP treatment compared with day 7 [33]. Therefore, we analysed microglial activation in the striatum and SNc at this time point. No significant differences between WT and Cx30 KO mice were observed in Iba1-positive cell numbers in either the striatum or SNc after NS or MPTP treatment (Fig. 10a, b), although MPTP caused vigorous activation of microglia in both locations on day 1 (Fig. 10c). Microglial activation was diminished by day 7 after MPTP administration to similar extents in WT and Cx30 KO mice (Fig. 10d).

**Fig. 11:**
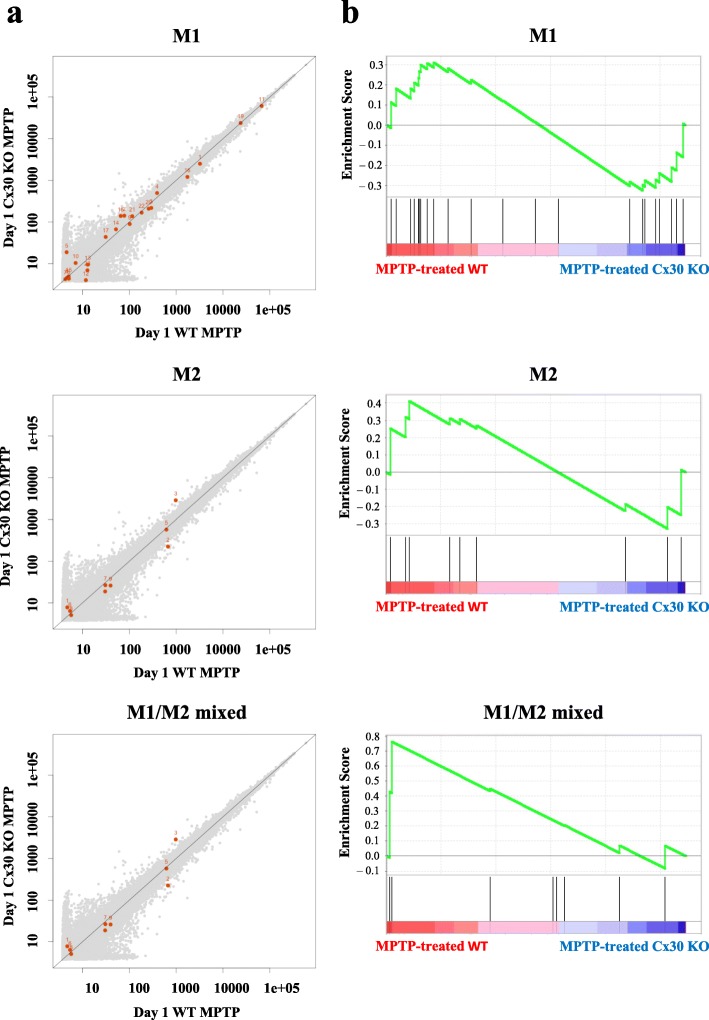
Microarray gene expression analysis of M1, M2, and M1/M2 mixed microglia gene sets at 1 day after MPTP treatment. **a** Scatter plots. The *X*- and *Y*-axis values are log10 scale-normalised signals. **b** Enrichment plots (green curve) show the running sum of enrichment score (ES) for each gene set. The score at the peak of the plots is the ES for each gene set. The black bars show where the members of the gene set appear in the ranked list of genes. A predominance of black bars to the left or right side indicates that most genes are upregulated in MPTP-treated WT mice or MPTP-treated Cx30 KO mice, respectively

Depending on the predominance of secreted factors, microglia can be classified into classical (M1: proinflammatory) or alternative (M2: anti-inflammatory) activation phenotypes. Thus, we used gene expression microarrays to examine M1-related proinflammatory, M2-related anti-inflammatory, and mixed M1/M2-related chemokine/cytokine expression in the striatum on day 1 after NS or MPTP treatment [34]. However, we found no difference between WT and Cx30 KO mice in the expression of any chemokines/cytokines after MPTP injection (Fig. 11a and Additional file 1: Figure S13 and Table S4). GSEA also revealed no significant differences in M1, M2, or M1/M2 gene expression between WT and Cx30 KO mice (M1: ES = − 0.3246, normalised *p* = 0.7926, FDR = 0.7410; M2: ES = 0.4118, normalised *p* = 0.7222, FDR = 0.7258; M1/M2 mixed: ES = 0.7616, normalised *p* = 0.0755, FDR = 0.1043; Fig. 11b).

### Expression of axon guidance pathway-related genes is downregulated in the striatum of MPTP-treated Cx30 KO mice

From our gene expression analyses, we extracted genes that met the following conditions: (i) differentially expressed in the striatum of MPTP-treated Cx30 KO mice compared with NS**-**treated CX30 KO mice and (ii) not differentially expressed in the striatum of MPTP**-**treated WT mice compared with NS**-**treated WT mice on day 1. The resulting gene set was evaluated by DAVID functional annotation and pathway enrichment analysis, which revealed a total of 11 KEGG pathways that were significantly enriched in Cx30 KO compared with WT mice after MPTP treatment (Additional file 1: Table S5). The most highly enriched pathway was the axon guidance pathway (*p* = 0.00033), which includes netrins, ephrins, semaphorins, and their receptors (Additional file 1: Figure S14). Within this pathway, we found that ten genes were downregulated and four genes were upregulated, suggesting an overall downregulation of this pathway in the striatum of Cx30 KO mice compared with WT mice after MPTP treatment (Additional file 1: Table S6). This is consistent with the results shown in the scatter plots and heat map of differentially expressed genes detected by microarray analysis (Additional file 1: Figure S15). These results therefore suggest that loss of Cx30 KO may reduce the potential for axon regeneration and repair in the striatum after MPTP treatment.

## Discussion

The main findings of the present study are as follows: (1) Cx30 expression in astrocytes was markedly upregulated in the striatum and SNc 7 days after MPTP administration in WT mice. (2) Cx30 deficiency modestly increased basal Cx43 levels and MPTP-upregulated Cx43 levels on day 1 after administration, but not on day 7 after administration. (3) Cx30 deficiency accelerated MPTP-induced DA neuron loss but had no effect on MPP^+^ production or DAT intensity in the striatum. (4) Cx30 deficiency reduced the responses of A2 and pan-reactive astrocytes on day 1 after MPTP treatment and decreased the expression of *S100a10* mRNA, *Gdnf* mRNA, and GDNF protein levels in the striatum on day 1, but not day 7, after MPTP treatment. (5) Cx30 deficiency partly suppressed GFAP upregulation and astrogliosis in the striatum and SNc on day 7 after MPTP treatment but did not change microglial responses. These results suggest that the hypersensitivity of Cx30 KO mice to MPTP is mainly due to the alterations in the acute responses of astrocytes.

Upregulation of both Cx30 and Cx43 in WT mice upon MPTP treatment can be either neuroprotective or neurotoxic. Increased DA neuron loss in Cx30-deficient mice indicates that the expression and upregulation of Cx30 are beneficial in the acute MPTP PD model. It has been reported that Cx30, but not Cx43, can compensate for other Cxs in the hippocampus [35]. Indeed, although Cx30 KO mice showed increased basal expression of Cx43 in the striatum and showed increased upregulation of Cx43 at 1 day after MPTP treatment compared with WT mice, this was not sufficient to compensate for the absence of Cx30. In another PD model, Cx30 was upregulated in the striatum after treatment with 6-OHDA, whereas Cx43 was not [14]. Collectively, these observations suggest that Cx30 is mainly involved in astrocytic neuroprotection, at least in these neurotoxin-induced PD models. Because the deficiency of Cx30 did not interfere with the MPTP-induced increase in Cx43, Cx30-mediated neuroprotection likely occurs through a Cx43-independent mechanism.

Both Cx30 and Cx43 are abundant in the perivascular astrocyte foot processes [36, 37], while Cx30 is also a major Cx in astrocyte processes around neurons in the grey matter [37]. Cx30 thus appears to be a component of the astrocytic metabolic network, providing an activity-dependent intercellular pathway for the delivery of energy sources, such as glucose and lactate, from the blood vessels to neurons [14, 38, 39]. This is consistent with the fact that Cx30 ablation reduces energy trafficking [38]. MPTP induced marked perivascular upregulation of Cx30 in the striatum in our study, similar to the previous observations with 6-OHDA [14]. This indicates that the increased transfer of energy sources from the blood vessels to neurons via astrocyte Cx30 channels may be partly responsible for neuronal survival after exposure to neurotoxic compounds, such as by MPTP and 6-OHDA, which inhibit the mitochondrial respiratory chain [40, 41].

Non-channel functions of Cx30 might also contribute to enhanced neuronal survival after MPTP treatment. Many channel-independent functions of Cxs in cell growth, migration, apoptosis, and signalling have been reported [16, 42], including inhibition of DNA synthesis and subsequent effects on gene expression [42, 43]. We evaluated the effects of Cx30 deficiency on gene expression patterns in the striatum of MPTP-treated animals and found that upregulation of neuroprotective A2 and pan-reactive astrocyte genes was markedly attenuated in Cx30 KO mice compared with that in WT mice. Upregulation of A2 astrocyte gene expression in WT mice in response to MPTP was similar to that observed in a brain infarct model [6]. Because neuronal injury by MPTP and acute ischaemia by middle cerebral artery occlusion both involve acute energy failure [44], it seems reasonable to propose that both insults predominantly induce A2 astrocyte gene expression. Attenuation of the neuroprotective A2 astrocyte response by Cx30 deficiency may well contribute to MPTP hypersensitivity in the Cx30 KO mice.

In murine MPTP models, dopaminergic terminal loss occurs earlier and to a greater extent than cell body loss [23, 45], as seen in human PD pathology [13]. We detected no significant difference in DAT intensity between WT and Cx30 KO mice, indicating that Cx30 deficiency does not upregulate DAT expression in DA neuron terminals. MPP^+^ concentrations in the striatum were also not significantly different in the two mouse strains, suggesting that MPP^+^ uptake by DA neuron terminals, and the subsequent damage is comparable in the presence and absence of Cx30. MPP^+^ is produced from MPTP in astrocytes and reduces their viability in a concentration-dependent manner [46]. It is possible that Cx30 KO astrocytes may be more vulnerable than WT astrocytes to MPP^+^. Since striatal astrocytes reportedly have protective functions on DA neuron terminals [47, 48], such heightened vulnerability may reduce the ability of Cx30 KO astrocytes to protect DA neurons. Our pathway enrichment analysis identified alterations in the expression of genes related to the axon guidance pathway, including netrins, ephrins, semaphorins, and their receptors. These molecules not only regulate axon guidance during brain development but also are involved in axon regeneration after brain injury in adults [49]. DA axon regeneration occurs in the striatum after MPTP or 6-OHDA treatment [50, 51], and ephrin signalling has been reported to influence DA neurogenesis in adult PD animal models [52, 53]. Multiple single nucleotide polymorphisms in axon guidance pathway genes are known to confer PD susceptibility [54]. Since astrocyte-secreted proteins and signals, such as netrins, ephrins, and semaphorins, act as axon guidance cues [55], altered expression of these genes may reduce the recovery from dying-back degeneration of DA neurons in the striatum of MPTP**-**treated Cx30 KO mice. Further studies will be required to clarify this issue.

We found that MPTP induced upregulation of the A2 astrocyte-related gene *S100a10* on day 1 after treatment in both WT and Cx30 KO mice, but the magnitude of the response was smaller in the Cx30 KO mice. However, there were no genotype- or treatment-related differences in *S100a10* expression at day 7 after MPTP administration, suggesting that the acute upregulation of *S100a10* may be beneficial for neuronal survival. S100A10 is a member of the S100 protein family and is expressed in numerous cell types, including astrocytes [6]. In the present study, we confirmed the expression of *S100a10* mRNA in striatal astrocytes by ISH. Although the precise functions of astrocytic S100A10 remain to be established, its functions may be similar to the non-channel functions of Cx30 [56–58]. S100A10 is required for membrane repair [56], cell proliferation [57], and inhibition of cell apoptosis by interaction with a Bcl-xL/Bcl-2-associated death promoter [58]. Thus, it is possible that S100A10 may promote the survival and proliferation of astrocytes upon MPTP exposure, thereby supporting the survival of neurons through secretion of neuroprotective factors. This hypothesis is supported by the observation that the number of GFAP-positive astrocytes was significantly decreased in Cx30 KO mice compared with WT mice at 7 days after MPTP treatment.

One such astrocyte-protective molecule is GDNF. Although *Gdnf* is not listed in the A2 astrocyte-related genes, GDNF is constitutively expressed by striatal astrocytes [31, 47] and is indispensable for DA neuron survival in adulthood [48]*.* Thus, we asked whether Cx30 deficiency might influence astrocytic-protective functions by molecules other than those reported to be A2 astrocyte-related genes. We found that basal levels of *Gdnf* mRNA and GDNF protein in the striatum were similar in WT and Cx30 KO mice, but their levels were significantly lower in Cx30 KO mice than in WT mice on day 1 after MPTP treatment. This acute reduction in striatal GDNF could be one reason for decreased DA neuron survival in MPTP-treated Cx30 KO mice. We confirmed the expression of *Gdnf* mRNA in astrocytes by ISH, consistent with previous reports in the 6-OHDA PD model [31]. Since we also found that GDNF was expressed in other cell types in the striatum, it is possible that Cx30 loss may also influence GDNF synthesis in those cells, perhaps as a consequence of the hyporeactivity of A2 astrocytes. One of the pan-reactive astrocyte genes that showed no change in the expression in response to MPTP in Cx30 KO mice was *Cp*, which encodes ceruloplasmin. This protein has properties similar to those of GDNF, including constitutive expression in the striatum [59]. In addition, ablation of ceruloplasmin leads to nigrostriatal degeneration, and its supplementation restores DA neurons [60]. Based on these observations, we suggest that Cx30 deficiency in astrocytes reduces constitutive expression of *Gdnf* and other survival factor genes, directly in striatal astrocytes and indirectly in neurons, which promotes the death of DA neurons.

Activated microglia found in the PD striatum and SNc are proposed to play an important role in neuroinflammation, which accelerates disease progression [61]. In our acute MPTP PD model, we observed widespread microglial activation in the nigrostriatal system. However, no difference was detected in microglia numbers or in M1- and M2-related gene expression between WT and Cx30 KO mice at 1 day after MPTP administration, when the microglial response was at its peak. Therefore, A2 astrocyte and pan-reactive astrocyte responses in the striatum may be more critical than the microglial response for ‘dying-back’ cell death of DA neurons in the SNc, at least in acute neurotoxin PD models. The contributions of astroglia and microglia to DA neuron loss in chronic PD models, however, remain to be determined. In our hands, Cx30 KO mice displayed attenuated A2 astrocyte-related *S100a10* upregulation and constitutive GDNF production on day 1, but not on day 7, after MPTP treatment, whereas Cx30/Cx43 upregulation was markedly enhanced at 7 days after MPTP administration in WT mice. Cx43 was also strongly upregulated 7 days after MPTP administration in Cx30 KO mice. We therefore suggest that *Gdnf*, *S100a10*, and other A2 astrocyte-related gene products may act as protective factors at a very early stage of MPTP exposure, while astroglial Cx channels may play roles at later times.

This study has several limitations. First, we examined the striatum and SNc only on days 1 and 7 after MPTP administration. Thus, investigation of additional time points may clarify the precise times at which neuroprotective factors operate. Second, we assessed differential gene and protein expression using whole dissected striatal tissues and not purified astrocytes. Although we confirmed that *Gdnf* and *S100a10* mRNA are expressed in striatal astrocytes, a quantitative comparison of GDNF and S100A10 expression in astrocytes, neurons, and other cell types will be informative. Third, we did not examine the channel functions of striatal Cxs in situ. Finally, we used an acute MPTP treatment regimen, whereas DA neuronal degeneration in PD patients occurs over a much longer time frame. However, because axonal degeneration precedes cell death even after acute MPTP treatment [23, 45], we believe that our results provide important insights into the role of astrocytes in dying-back degeneration of DA neurons in PD.

## Conclusions

The present study indicates that Cx30 deficiency attenuates the neuroprotective functions of astrocytes in the striatum following a neurotoxic insult that causes acute energy failure, thereby amplifying DA neuron loss due to dying-back degeneration. Therefore, we propose that the augmentation of Cx30 functions could be a potential therapeutic strategy for PD patients.

## Additional file

Additional file 1: **Figure S1.** Western blots of Cx30 on day 1. **Figure S2.** Western blots of Cx30 on day 7. **Figure S3.** Effects of MPTP on Cx30 expression in SNc. (a, b) Upper panels: triple immunostaining on days 1 (a) and 7 (b). Lower panels: enlarged images inwhite boxes. Scale bars: upper panels, 100 μm; lower panels, 50 μm. **Figure S4.** Cx30 (green) and GFAP(red)immunostaining in striatum and SN. Scale bars: 100 μm. **Figure S5.** Effects of MPTP on Cx43 expression in SNc. Left two columns: triple immunostainingon days 1 (a) and 7 (b). Right two columns show enlarged images inwhite boxes. Scale bars: left two columns, 100 μm; right two columns, 50 μm. **Figure S6.** Effects of MPTP on Cx43 expression. (a, b) Number of Cx43-immunoreactive dots. (c, d) RT-PCR analysis of Cx43 mRNA levels. (e, f) Western blot analysis of Cx43 protein levels. Blot images in Figure S2. **Figure S7.** Western blots of Cx43 on days 1 and 7. **Figure S8.** Western blots of Cx43 on day 7. **Figure S9.** MPP+ concentration. **Figure S10.** DAT immunostaining (a) and quantification of DAT-positive fibre density (b). Scale bar: 50 μm. **Figure S11.** Heatmap of A1, A2, and pan-reactive astrocyte gene expression. **Figure S12.** Western blotsof GFAP. **Figure S13.** Heatmap of M1, M2, and M1/M2 mixed microglia gene expression. **Figure S14.** KEGG pathway map of the axon guidance pathway. **Figure S15.** Microarray expression dataofaxon guidance pathway-related genes. (a) Scatter plots. (b) Heatmap. **Table S1.** Antibodies used for immunohistochemistry. **Table S2.** Primers used for ISH. **Table S3.** Gene names indicated by red dots in Fig. 6. **Table S4.** Gene names indicated by red dots in Fig. 11. **Table S5.** KEGG pathway analysis of microarray data. **Table S6.** Gene names indicated by red dots in Additional file 1: Figure S15a. (DOCX 100840 kb)

## Abbreviations

6-OHDA: 6-Hydroxydopamine
BDNF: Brain-derived neurotrophic factor
Cx: Connexin
DA: Dopaminergic
DAT: Dopamine transporter
DOPAC: 3,4-Dihydroxyphenylacetic acid
ELISA: Enzyme-linked immunosorbent assay
ES: Enrichment score
FDR: False discovery rate
GDNF: Glial cell-derived neurotrophic factor
GFAP: Glial fibrillary acidic protein
GSEA: Gene set enrichment analysis
HPLC-ECD: High-performance liquid chromatography with electrochemical detection
HVA: Homovanillic acid
Iba1: Ionised calcium-binding adapter molecule 1
ISH: In situ hybridisation
KO: Knockout
LC-MS/MS: Liquid chromatography-tandem quadrupole mass spectrometry with electrospray ionisation
MPTP: 1-Methyl-4-phenyl-1,2,3,6-tetrahydropyridine
NS: Normal saline
PBS: Phosphate-buffered saline
PD: Parkinson’s disease
RT-PCR: Reverse transcription polymerase chain reaction
SN: Substantia nigra
SNc: Substantia nigra pars compacta
SNr: Substantia nigra pars reticulata
TBST: Tween-20 in Tris-buffered saline
TH: Tyrosine hydroxylase
WT: Wild-type
