# Supplementary material for: Connexin 30 deficiency attenuates A2 astrocyte responses and induces severe neurodegeneration in a 1-methyl-4-phenyl-1,2,3,6-tetrahydropyridine hydrochloride Parkinson’s disease animal model
Source: J Neuroinflammation. 2018 Aug 13;15:227. doi: 10.1186/s12974-018-1251-0 (PMC6090688; doi:10.1186/s12974-018-1251-0)
Supplement: Supplementary file 1 — Figure S1. Western blots of Cx30 on day 1. Figure S2. Western blots of Cx30 on day 7. Figure S3. Effects of MPTP on Cx30 expression in SNc. (a, b) Upper panels: triple immunostaining on days 1 (a) and 7 (b). Lower panels: enlarged images inwhite boxes. Scale bars: upper panels, 100 μm; lower panels, 50 μm. Figure S4. Cx30 (green) and GFAP(red)immunostaining in striatum and SN. Scale bars: 100 μm. Figure S5. Effects of MPTP on Cx43 expression in SNc. Left two columns: triple immunostainingon days 1 (a) and 7 (b). Right two columns show enlarged images inwhite boxes. Scale bars: left two columns, 100 μm; right two columns, 50 μm. Figure S6. Effects of MPTP on Cx43 expression. (a, b) Number of Cx43-immunoreactive dots. (c, d) RT-PCR analysis of Cx43 mRNA levels. (e, f) Western blot analysis of Cx43 protein levels. Blot images in Figure S2. Figure S7. Western blots of Cx43 on days 1 and 7. Figure S8. Western blots of Cx43 on day 7. Figure S9. MPP+ concentration. Figure S10. DAT immunostaining (a) and quantification of DAT-positive fibre density (b). Scale bar: 50 μm. Figure S11. Heatmap of A1, A2, and pan-reactive astrocyte gene expression. Figure S12. Western blotsof GFAP. Figure S13. Heatmap of M1, M2, and M1/M2 mixed microglia gene expression. Figure S14. KEGG pathway map of the axon guidance pathway. Figure S15. Microarray expression dataofaxon guidance pathway-related genes. (a) Scatter plots. (b) Heatmap. Table S1. Antibodies used for immunohistochemistry. Table S2. Primers used for ISH. Table S3. Gene names indicated by red dots in Fig. 6. Table S4. Gene names indicated by red dots in Fig. 11. Table S5. KEGG pathway analysis of microarray data. Table S6. Gene names indicated by red dots in Additional file 1: Figure S15a. (DOCX 100840 kb) [file 12974_2018_1251_MOESM1_ESM.docx]

**Additional Figure S1**

**Additional Figure S2**

**Additional Figure S3**

Cx30 / GFAP / DAPI

**a**

**NS**

**MPTP**

**Day 1 SNs**

**b**

**NS**

**MPTP**

**Day 7 SNs**

**SNc**

**SNc**

**SNc**

**SNc**

**SNr**

**SNr**

**SNr**

**SNr**

**Additional Figure S4**

**Day 7 NS**

**WT**

**Striatum**

**SN**

**Cx30 / GFAP**

**SNc**

**SNc**

**SNr**

**SNr**

**SNc**

**SNr**

**Day 7 NS**

**Cx30 KO**

**Day 7 MPTP**

**Cx30 KO**

**Additional Figure S5**

Cx43 / GFAP / DAPI

**WT**

**Cx30 KO**

**a**

**Day 1 SNs**

**WT**

**Cx30 KO**

**WT**

**Cx30 KO**

**b**

**Day 7 SNs**

**WT**

**Cx30 KO**

**SNr**

**SNr**

**SNr**

**SNr**

**SNr**

**SNr**

**SNr**

**SNr**

**SNc**

**SNc**

**SNc**

**SNc**

**SNc**

**SNc**

**SNc**

**SNc**

**NS**

**MPTP**

**NS**

**MPTP**

**Additional Figure S6**

**Additional Figure S7**

**Additional Figure S8**

**Additional Figure S9**

**Cx30 KO**

**WT**

**MPP^+^ (ng / mg tissue)**

**N.S.**

WT	Cx30 KO

MPTP

**Additional Figure S10**

**Cx30 KO**

**WT**

**N.S.**

WT	Cx30 KO

MPTP

**a**

**b**

**Additional Figure S11**

**WT**

**Cx30 KO**

**WT**

**Cx30 KO**

**NS**

**MPTP**

**-2**

**-1**

**0**

**2**

**1**

**Distance from median**

**Color key**

***Cd44***

***Timp1***

***S1pr3***

***Vim***

***Gfap***

***Cxcl10***

***Aspg***

***Osmr***

***Steap4***

***Lcn2***

***Serpina3n***

***Cp***

***Hspb1***

**Pan-reactive**

**A1**

***Fkbp5***

***Srgn***

***Ggta1***

***Ligp1***

***Psmb8***

***Gbp2***

***H2-t23***

***H2-d1***

***Amigo2***

***Serping1***

***Fbin5***

**A2**

***Clcf1***

***Tgm1***

***Ptx3***

***S100a10***

***Sphk1***

***Slc10a6***

***Ptgs2***

***Emp1***

***Cd14***

***B3gnt5***

***Tm4sf1***

***Cd109***

**Additional Figure S12**

**Additional Figure S13**

**M1**

**M2**

**WT**

**Cx30 KO**

**WT**

**Cx30 KO**

**NS**

**MPTP**

**-2**

**-1**

**0**

**2**

**1**

**Distance from median**

**Color key**

**M1/M2 mixed**

***Il17f***

***Csf2***

***Cxck10***

***B2m***

***Ccl2***

***Spp1***

***Ccl3***

***Cxcl16***

***Ccl19***

***Tnf***

***Tnfsf10***

***Mif***

***Lta***

***Il12b***

***Ltb***

***Ccl5***

***Il15***

***Hc***

***Hd70***

***Il18***

***Tnfsf13b***

***Hsp90ab1***

***Adipoq***

***Il1rn***

***Lif***

***Il16***

***Ccl17***

***Ccl12***

***Ccl24***

***Csf1***

***Il13***

***Vegfa***

***Il21***

***Il27***

***Osm***

***Ppbp***

***Csf3***

***Ifna2***

***Pf4***

***Ifng***

**Additional Figure S14**

Axon outgrowth

Axon repulsion

Axon attraction

Axon Guidance

**Additional Figure S15**

**Supplementary Table S1. Antibodies used for immunohistochemistry.**

**Supplementary Table S2. Primers for *in situ* hybridisation**

**Supplementary Table S3. Gene names indicated red dots in Figure 6.**

**Supplementary Table S4. Gene names indicated red dots in Figure 11.**

**Supplementary Table S5. KEGG pathway analysis of microarray data.**

**Supplementary Table S6. Gene names indicated red dots in Supplementary Figure S15** **a.**
